# Collectanea

**Published:** 1833-01

**Authors:** 


### COLLECTANEA.

Floriferis ut apes in saltibus omnia libant,
Omnia nos, itidem, depascimur aurea dicta.

### PATHOLOGY.

*Curious Account of Phthisis caused by Mental Depression.* AMONG the occasional causes of phthisis, I know of none of more assured operation than the depressing passions, particularly if strong and of long continuance; and it is worthy of remark, that it is the same cause which seems to contribute most to the development of cancers, and all the other accidental productions which are not analogous to any of the natural tissues. This is, perhaps, the only cause of the greater frequency of consumption in large cities. In these, the single circumstance of the inhabitants having more numerous relations with one another, is in itself a cause of more frequent and deeper vexation; while the greater prevalence of immorality of every kind is a constant source of disappointment and misery, which no kind of consolation, and not even time itself, can effectually remove. I had under my own eyes, during a period of ten years, a striking example of the effect of the depressing passions in producing phthisis; in the case of a religious association of women of recent foundation, and which never received from the ecclesiastical authorities any other than a provisional toleration, on account of the extreme severity of its rules. The diet of these persons was certainly very austere, yet it was by no means beyond what nature could bear. But the ascetic spirit which regulated their minds was such as to give rise to consequences no less serious than surprising: not only was the attention of these women habitually fixed on the most terrible truths of religion, but it was the constant practice to try them by every kind of contrariety and opposition, in order to bring them as soon as possible to an entire renouncement of their own proper will. The consequences of this discipline were the same in all: after being one or two months in the establishment, the catamenia became suppressed, and in the course of one or two months thereafter phthisis declared itself! As no vow was taken in this society, I endeavoured to prevail upon the patients to leave the house as soon as the consumptive symptoms began to appear, and almost all those who followed my advice were cured, although some of them exhibited well-marked indications of the disease. During the ten years that I was physician of this association, I witnessed its entire renovation two or three different times, owing to the entire loss of all its members, with the exception of a small number, consisting chiefly of the superior, the gatekeeper, and the sisters who had the charge of the garden, kitchen, and infirmary. It will be observed that these individuals were those who had the most constant distractions from their religious tasks, and that they also went out pretty often into the city, on business connected with the establishment. In like manner, in other situations it has appeared to me that almost all those who became phthisical, without being constitutionally predisposed to the disease, might attribute the origin of their complaint to grief, either very deep or of long continuance. —FORBES' *Translation of* LAENNEC *on Diseases of the Chest.*

A parallel instance of phthisis being *caused*, or its *latent causes* being aroused, by violent depressing passions, will be in the recollection of all as occurring in the case of the late lamented MR. ROSE, surgeon to St. George's hospital.—ED.

### PRACTICAL MEDICINE.

*Ptyalism cured by Opium;* by Dr. J. Graves. A middle-aged woman, of delicate appearance, applied to me for advice on the 26th of December last. She had laboured under a profuse and long-continued leucorrhœa, which ceased rather suddenly in the beginning of September, and was followed by a slight degree of anasarca. This disappeared under a course of diuretic and purgative medicines, but she remained in a debilitated state, and experienced much distress from irritability of stomach, and finally from obstinate retching. In October this symptom also suddenly subsided, and was succeeded by a remarkable and profuse salivation, which continued unabated, notwithstanding the use of various purgatives and astringent medicines, gargles, &c.

In twenty-four hours she spits more than a pint and a half of fluid, consisting of a whitish, viscid mucus, secreted by the mucous membrane of the fauces and back of the pharynx, from whence it is thrown into the mouth by a *hawking*, renewed every two or three minutes, with scarcely any interruption either during the night or day, and rendering the patient truly miserable from want of sleep. The throat and fauces are pale, and their soft parts extremely flabby and relaxed, although there is constant irritation in the throat, in consequence of the presence of an unnatural quantity of mucus; yet no soreness is felt, neither do the parts appear inflamed. The salivary glands are not concerned in the disease, and do not secrete more than the usual quantity of fluid. Her appetite is very bad, her skin dry, and she has a haggard, emaciated countenance.

The well-known good effects of opium in several diseases of increased secretion, diabetes, diarrhœa, and certain forms of dropsy, suggested to me the trial of this medicine in the apparently almost hopeless case I have related, and I accordingly ordered the patient one grain of opium every fourth hour. On the following day, she returned to inform me that she had slept during the whole night, and on awaking had no return of the spitting. Her joy was great, and she and her friends considered the effect of the pills, in thus suddenly stopping the spitting, as most extraordinary; and I must confess that my surprise was almost equal to theirs. She then told me that several medical students, who lived in her house, and who had witnessed the previous violence and obstinacy of her complaint, had been so much struck by its sudden cessation under the influence of the pills, that she was commissioned by them to inquire what I ordered. I mention this circumstance, to shew how very remarkable was the benefit she received from the opium. The pills were continued for some days, when the quantity of opium was augmented on account of some recurrence of the spitting: unfortunately they induced constipation of bowels, and, consequently, she has been frequently obliged to leave them off; but she is, on the whole, much improved in health, and although she is still subject to the disease, its severity is comparatively trifling, and it uniformly disappears almost entirely when she recurs to the use of opium. She visited me this day, the 18th of March. In connexion with this subject I may observe, that a lady, a patient of mine, who took a large quantity of mercury many years ago, has been ever since subject to occasional returns of salivation, in every respect resembling that produced by mercury. During these fits, which are always brought on by exposure to cold, the mouth is sore; the gums red and swollen; the salivation copious, and the breath is strongly impregnated with the mercurial fetor. Several such cases have been recorded by others, and are extremely instructive, proving that the deleterious effects of mercury on the system may lie long dormant, and be afterwards suddenly called into action by various causes. In this way we may explain the attacks of periostitis, to which persons who have been mercurialized are often liable for years, upon exposure to cold. Formerly such attacks were indiscriminately attributed to a remnant of the syphilitic taint, but I have witnessed the same occurrence in persons who had no syphilitic disease, and who had taken mercury for other complaints. One of the most violent attacks of periostitis I ever witnessed, followed salivation in a lady to whom mercury had been given several months before, for the cure of peritonitis. To conclude, I have seen many cases totally at variance with the assertion of Ballingall and others, that the secondary symptoms, attributed by recent authors to mercury, are owing to lues; not being observed, as they state, except in cases where that medicine had been exhibited for the cure of the venereal disease. The venereal poison produces a peculiar train of primary and secondary symptoms; the mercurial another, and that not very dissimilar. Experience has proved that, in general, the venereal symptoms yield to the action of mercury; but occasionally this is not the case, in consequence either of some constitutional peculiarity, or an injudicious use of this mineral, and thus the constitution labours under a modified disease, resulting from the combined effects of the two poisons. Attention to the effects of the remedy in the first instance will generally prevent the occurrence of so disastrous a complication. My own experience has convinced me that the majority of venereal cases may be cured by the non-mercurial plan of treatment; but whenever the disease, whether primary or secondary, proves more than usually obstinate, the practitioner should then have recourse to mercury; the previous treatment will render its curative influence at once safer and more energetic.—*Dublin Journ. of Med. and Chem. Sciences.*

---

### PRACTICAL SURGERY.

*Lithotripsy.* We have watched the improvements in the means of destroying calculi in the human bladder with extreme anxiety, and have, on several former occasions, laid before our readers detailed accounts of the various modifications which the instruments and the operation have already undergone. Very much pleased were we with the three-branched instruments of Leroy and Civiale; and still more so with the four-branched instrument of Heurteloup, the claws of which are severally moveable, as well as with his two-branched "brise-coque;" but, beautiful as these are in principle, in practical applicability, they are all far, very far, surpassed by the last-named gentleman's "Percussor," which, since we last mentioned it, as being exhibited at the Royal Institution, has undergone much improvement, and been made much stronger. Other instruments have lately been constructed on the same principle with that of the Baron's; and one of them having been submitted to the consideration of the Westminster Medical Society, (by Mr. Costello,) Baron Heurteloup was requested by the Society to allow his apparatus to be examined, and to show publicly his mode of operating; which he immediately agreed to in the most handsome manner. The following is the short memoir with which the demonstration was prefaced, and which we have much pleasure in being able to give to our readers.—Editors.

Mr. President and Gentlemen:

Since I applied the *system of percussion* to the comminution of calculi in the interior of the bladder, by means of a curved instrument and hammer, called *percuteur courbe à marteau*, many persons have attempted to construct a similar apparatus; some have even sought to modify the Percuteur, and seemed to forget that, without performing numerous and varied experiments, and, above all, without operating frequently upon the living subject, we cannot arrive at giving to surgical instruments a proper and advantageous construction. Hence we have seen produced a vast number of defective instruments, and their imperfections have been brought forward as useful modifications, before surgeons whose other avocations, or indifference to the question, have prevented them from thoroughly investigating the subject.

You will readily conceive, Gentlemen, how unpleasant and useless it would be to reply to, and enter into discussion with, the numerous individuals who think they have advantageously modified my instruments.

For about eight years that I have been labouring to present lithotripsy to the scientific world, as an art susceptible of extensive development, and not as a naked and barren subject, I have so frequently seen my combinations imperfectly imitated, or rendered defective by additions and subtractions, that it has been a matter of necessity for me to lay down as an invariable rule, never to notice any such modifications, until they really merited attention, in consequence of their utility being sanctioned and proved by surgical facts.

I have no reason to repent having done so, since all these modifications have of themselves fallen into oblivion, because none of them have ever been employed with success in the relief of patients labouring under stone in the bladder. Thus, you, perhaps, have heard of modifications of the *brise coque*, with which fragments of calculus are pulverized; of the *four-branched instrument*, with which large spherical calculi are excavated, before they are acted upon by the hammer, &c.; but, I think, none have ever heard a patient afflicted with this serious malady attribute his complete recovery to any of these pretended improvements.

You will not, therefore, be surprised, Gentlemen, if, on the present occasion, I remain silent respecting any modifications in an instrument of which I am the inventor, until the advantages of these modifications are established by practical and well-supported facts. A person who entertains the idea of making any alteration in an instrument which has been repeatedly employed with success, must found this idea on practical experience, and not on the caprice of too ardent an imagination. When I modified the first models of the *percuteur*, I was not guided by my taste or fancy, but by study, numberless experiments, and repeated operations on the living subject. It is by these experiments and operations that my eyes were opened to imperfections which were to be carefully avoided, and that I was consequently directed to a path of improvement. Hence sprang a long series of experiments, some of the results of which I here lay before you, in order that you should duly appreciate an instrument constructed with so much labour and care, and the advantages of which have already been sanctioned and illustrated by upwards of eighty successful applications on the living subject. If you examine these instruments, you will find it easy to follow the series of numerous improvements which were pointed out to me, and you will also perceive the imperfections of those which were first constructed. After having examined in a surgical, and above all in a philosophical point of view, you will, I feel assured, blame those who took pleasure in spreading abroad unfounded statements of an operation in which one of the earliest of these instruments yielded. I am alluding to the case of Colonel Rawken, with which you must be well acquainted, for it has been much more spoken of than all my successful cases put together. The two branches or curved portion of the percuteur remained a little apart, and it became necessary, in order to close them and withdraw the instrument, to make a small incision into the urethra, such as are so frequently practised in the extraction of small calculi lodged in this passage. A case so simple in itself gave rise to the circulation of reports of the most serious nature. It was untruly said, that the operation of lithotomy was performed in order to extract the instrument from the bladder; and it was as untruly added, that the patient died immediately, or very shortly afterwards, in consequence of the operation; whilst, in truth, lithotomy was performed, not because any thing that was previously done rendered it indispensably necessary, since the instrument was easily withdrawn by the means above mentioned, but because it was considered the most prudent measure to adopt. The patient, who was an old man of seventy-five, died, not directly after the operation, but nine weeks after, and expired through exhaustion and want of strength to rally; for at the time of his death the wound was nearly healed, and almost all the urine flowed out naturally by the urethra.

This misrepresented fact, Gentlemen, constituted the grounds on which was founded the condemnation of the *percuteur courbe;* as if an invention, just springing into existence, ought at once to be rejected, for no better reason than the imperfections which may be displayed in its earliest infancy. If steam-vessels had been thrown aside, and the idea discarded, because, in the first applications of this powerful agent, a vessel was blown up, would it not have been acting a most unreasonable part, and should we not have abandoned one of the most sublime inventions of the human mind? If you reflect a moment, Gentlemen, you will find these remarks just, and will readily conceive that it is only by long experience that the instruments which I now present to you were rendered advantageously applicable, and that it is only by following the same arduous route, and thus acquiring useful lessons, that others should consider themselves competent to modify them. As a proof of this, the case just alluded to induced me to make the most minute researches as to the best quality of metal to select, the most favorable manner of developing great power in the small instruments which the urinary organs compel us to employ; the size and weight of the hammer, and the different lengths of the lever; the best manner of dealing the blows, and, in a word, a vast number of particular circumstances, which, by close study and observation, have now rendered the system of percussion one of the safest and most efficient of the lithotriptic means.

After these general remarks on the little importance to be attached to modifications unsupported by successful facts, it only remains with me to give you some account of my own labours. I am among you, not for the purpose of entering into controversies, which I am ever anxious to avoid, but as a foreigner proud and happy to have been called upon by you to appear, and most willing to give you the informations that you may require on lithotripsy, and more especially on the *percuteur courbe.*

The early history of lithotripsy is familiar to all; it would, therefore, be a work of supererogation for me to pretend to afford information to such an enlightened audience: suffice it to say, that, as the result of the meritorious exertions of Gruithuisen, Elderton, Amussat, Civiale, Leroy, and others, lithotripsy, in 1824, was performed by means of an instrument possessing the properties of grasping calculi of moderate dimensions, and boring a hole in them every time the surgeon succeeded in seizing them. This was the three-branched instrument invented by Leroy, and in some points usefully modified by Civiale.

This instrument, tolerably well calculated to relieve patients whose bladders contained small spheroidal stones, I found inefficient and inapplicable when there existed larger calculi, which required to be bored repeatedly before they gave way, and consequently a too frequent repetition of the manipulations was necessary.

To remedy the disadvantages of having to make so many perforations in a calculus, I invented an instrument by which the stone, when seized, was at once hollowed out like an eggshell. This shell was much more easily comminuted, either by the instrument already in use, or by any other that might be found more applicable. With this excavating apparatus, the calculus was more efficiently acted upon, and more readily broken, in proportion to its greater degree of sphericity. The concave portions of stone resulting from the excavation were, however, with difficulty broken down with either of these two instruments: this induced me to construct the *brise coque,* which, by means of two strong branches, of which it consisted, rapidly comminuted these flat and concave fragments.

By means of these three instruments, it was possible to comminute small stones; to excavate, and sometimes break down, large spheroidal ones, and to pulverise the fragments with rapidity. But even these means were found inefficient in cases of flat and oval calculi of considerable size, which are so often met with in the human bladder; and also in those cases where there was still to break the shell of a large and very hard spheroidal calculus, which had been before excavated. It was therefore necessary to search still further: in this I was materially assisted by the experience I had acquired, and the result of my studies was the discovery of the system of percussion and the *curved percuteur à marteau*.

About six years back, whilst operating publicly upon a patient with the excavating apparatus, at the Hôtel Dieu, in Paris, just as the stone was seized and the perforator was about to be brought into action, I perceived that I had forgotten the pulley. Finding that the drill bow was of no use, it struck me that, since the instrument was firmly maintained by the fixed point, sharp blows might with perfect safety be directed, by means of the perforator, against the stone, which might thus be comminuted. This was accomplished, and the instrument was then closed and withdrawn. The next day the patient voided a much larger quantity of detritus than could have been expected from so simple a manœuvre.

Such, Gentlemen, is the first idea I had of percussion.

In another operation performed publicly about two years ago at the Greenwich hospital, I, for some time, tried in vain to break down a very hard spherical calculus which had been excavated. The stone being much weakened by being hollowed out, it struck me that it might be broken with a three-branched instrument, if, instead of merely applying its boring action, I could devise the means of directing against the calculus, through the medium of the drill, the powerful shock of a hammer. This was accomplished by adapting to the instrument this particular piece, which was called a support, and was intended to prevent the blows of the hammer from causing the internal tube to slide up the external canula, and thus occasion the expansion of the branches and the dropping of the stone from their grasp. (The support adopted to the three-branched instrument was here presented and explained.)

By so disposing the instrument, the stone was completely broken; but not without putting the patient to considerable pain, for being badly supported by the hooked extremities of the branches, it communicated more or less to the bladder, the vibration resulting from the blows of the hammer. It was therefore necessary to discover some other means.

Such is the second fact, which still more forcibly than the former induced me to turn my attention to the *system of percussion*.

Finally, nearly two years ago, in an operation performed upon a reverend gentleman, whose bladder contained several small calculi, I employed the three-branched instrument, but replaced the drill bow, by the application of a hammer. It was evident that this was not only a more rapid method of proceeding than the slow and gradual boring action, but that the blows of the hammer reduced the stone to a soft gravelly sand, instead of forming a very fine powder, which is liable to adhere to the coats of the bladder, and be evacuated with difficulty.

This third fact tended to convince me still more thoroughly that percussion might be most advantageously applied to lithotripsy, and pointed out to me still more directly the necessity of finding suitable means of bringing this system into practice.

Large spheroidal calculi, by presenting almost equal radii, appeared in perfect relation to an instrument destined to excavate them by a rotatory movement; but flat and oval calculi, by their great inequality of radii, were proportionately ill adapted to this system. In the same manner as the physical properties of spherical calculi gave rise to the idea of acting upon them by excavation, so the shape of flat and oval calculi clearly indicated the advantages to be derived from applying to their comminution the system of percussion. All flat and oval calculi present two surfaces, and it was evident that, in order to seize and hold them well, the instrument constructed for that purpose must consist of two opposed planes, which could only be obtained by means of two branches.

As the necessity of seizing and holding flat and oval stones required an instrument consisting of two branches, so the necessity of comminuting these stones required that these branches should have a curved direction: for, to act upon a stone by percussion in the interior of the bladder, the stone must be firmly supported by an immoveable plane, whilst a moveable one is forcibly propelled against it. But, in order to effect this in the human bladder, where the size of the instrument is limited to three or four lines, and where we are compelled to transmit the force of percussion through the entire length of the urethra, it was indispensably necessary that the planes retaining the calculus should be perpendicular to the straight portion of the instrument which remains in the urethra. Hence we see the absolute necessity of giving a curve to the instrument.

Thus it will, I think, appear evident that previous observations furnished me with the idea of developing in an instrument the powerful system of percussion; and that logical deductions induced me to make it consist of two branches, and give these branches a curved direction, rather than a desire to imitate other instruments, which were not even destined to act by percussion.

These were the principles on which I constructed the curved *percuteur*, and, if you examine it, you will find that it consists of two planes, which, by means of a curve, become perpendicular to that straight portion of the instrument which remains in the urethra. It is by bringing these planes together that a flat or oval calculus is seized and firmly held; and it is by propelling the moveable plane towards the immoveable one, by the active power of a hammer, that a calculus interposed between them is comminuted with the greatest facility. The stone is by these means reduced to a greater number of fragments, in proportion to the width of the planes.

You must still be well aware, Gentlemen, that, however perfect the instrument may be rendered, it would be of no sort of use without the assistance of one of my earliest inventions: I mean the rectangular bed, and the *point fixé* (fixed point), which was devised for giving perfect immobility to the instrument.

As was before mentioned, the *percuteur* was to present a moveable and an immoveable plane: well, it is by means of this fixed point or support, placed at the anterior part of the rectangular bed, that the perfect and indispensable immobility of the posterior curve of the instrument is obtained. It is this fixed point which allows the safe application of the power of a hammer to destroy calculi in the interior of the human bladder; a method of operating which, but two years back, was far from being looked upon as likely to contribute to the relief of one of the most serious maladies to which the bladder is liable.

This instrument completes the series of means by which I perform the operation of lithotripsy, and it was employed for the first time in London, on the 31st May, 1831, in presence of Sir Astley Cooper, Mr. Travers, and my friend Dr. Negri: and since this period I have, with this instrument, and before numerous members of the medical profession, relieved a sufficient number of patients to justify me in considering the *percuteur* one of the most valuable and efficient of all the lithotriptic agents hitherto employed.

In conclusion, Gentlemen, I have now only to show you the mechanical action by which the *percuteur* comminutes a calculus, and to beg the favor of the company of the president, and that of any other five or six gentlemen of the Society, (that being about the number that the patient would feel pleasure in seeing,) the day after to-morrow, at any hour that shall be convenient, in order to see the instrument employed on the living subject. I shall then, I hope, be enabled to give you a favorable and more complete idea of this new method of operating.

P.S. Twelve members of the Society, including the four presidents, having been invited, witnessed the Baron operate with his percussor. The calculus was seized with the greatest apparent ease, and the gratification expressed by all on *hearing* the stone crushed by the successive blows of the hammer, and subsequently *seeing* the fragments pass away by the urethra, was extreme. The stone was entirely destroyed in three or four sittings, altogether not occupying above twelve or fifteen minutes, and the patient is now quite well. He seemed to suffer very little uneasiness during the operation, and stated that he scarcely felt any thing of the vibrations produced by the hammer, and that passing the instrument gave him less pain than he had frequently suffered before from the introduction of the simple sound.

On Saturday, the 22d December, Baron Heurteloup presented, through Mr. Burnett, to the Westminster Medical Society, the patient referred to in the above report. He is entirely cured of his complaint; has no symptoms at all remaining; and he shewed to the members the fragments of the stone which had been comminuted by the percussor.

---

### MISCELLANEOUS.

### *Observations on Parturition, taken from a Lecture delivered at the Theatre of Anatomy and Medicine, Marsden street, Manchester, October* 3, 1832. By John Roberton, one of the Surgeons to the Lying-in Hospital.

It has always been the policy of those who decry man-widwifery to instance the ease and safety of parturition in brutes, and in women amongst savages; and thence to infer, that the same process in civilized society would be equally safe and easy, were it only left (as they contend it ought) to the efforts of nature, and the assistance of matrons. These objectors, it would appear, forget that the practice they recommend was universally followed, in every country in Europe, till little more than a century ago; and that it was gradually abandoned, apparently, through the influence of increasing humanity and intelligence.

The assumed safety of parturition in brutes, of which I shall speak first, involves considerable fallacy. In brutes, it is true, we discover a wonderful degree of perfection in the performance both of the organic and animal functions. But this can be said of them in the wild state only. In that state they rarely exhibit varieties in any respect; that is to say, they very rarely deviate from the primal type of the species to which they belong. In colour, form, habits, and what is of much importance in the present argument, size, they are produced the same in successive generations. In a herd of bisons, for example, amounting perhaps to many thousands, it is generally impossible to detect even a single instance of deviation, in regard to colour, from the natural dun. In our common domesticated animals, a similar uniformity of type is soon produced, when they are turned loose to breed in the wilderness. This is seen in the horses and cattle which the Spaniards, selecting from the various breeds of their own country, introduced into the savannas of the new world. There they are found, in vast herds, not, as in the domesticated state, of various colour, and size, but of a brown bay, a colour common to a great number of wild quadrupeds; and in other respects presenting the uniform features of *feræ naturæ.* These circumstances naturally lead us to infer that monstrosities, as well as diseases, must be unknown among wild animals, an inference near the truth; yet we shall err, in regard to this point, if we venture to generalise without a careful examination of facts; for although it be true that monstrosities and diseases are extremely rare in wild animals, various instances of both have been known, and, were our opportunities of observation greater, probably we should discover more. Camper, a high authority, assures us that he had in his possession specimens of malformation belonging to every species of animal: among others, a gazelle with two heads; also a serpent and a tortoise, each with two heads; and a lizard with the two hinder feet in one. In the great work of Daubenton, examples, I believe, are given of a similar kind. Of the diseases of animals, in their wild state, we are not likely to know much; yet we are not without a number of observations on this point, for which I refer you to the works of Camper. I will merely mention one instance, on the authority of Adair. In the year 1766 an epidemic malady prevailed among the wild beasts, particularly the deer, in the remote woods of West Florida. The Indians, in their winter's hunt, found several lying dead; some in a helpless condition, and others fierce and mad.

The condition of domesticated is extremely different from that of wild animals. No sooner are the natural habits of animals modified by the influence of man, than a great variety of changes rapidly ensues. Each particular species soon presents, within itself, remarkable diversities, in colour, instinct, figure, and size. They now become liable to numerous diseases; and exhibit likewise almost as great a variety of congenial imperfections as man himself. But of all the organic changes to which they are subject, none is more prominent and worthy of our attention than that which respects the generative system. Frequent sterility now succeeds to uniform fecundity, and abortion, in some species, becomes so frequent, under particular circumstances, that the disposition is even thought to be propagated by a specific contagion. Be this as it may, it often pervades an entire dairy: and is extremely difficult of remedy. So far, again, from bringing forth their young with uniform ease and safety, the mortality resulting from parturition, under certain circumstances which I shall specify, is incomparably greater than it is in our own species. And, even when the circumstances are of the most favorable kind, this act is attended with more or less pain, and, occasionally, with risk to life.

Without enlarging on the subject of comparative obstetrics, (although I must be permitted to say that I regard it as one of high interest to the student of midwifery,) it may be well if I make a few remarks, and state one important fact respecting it, derived from a practical person every way worthy of credit.*

Of course, those domesticated quadrupeds only which bring forth one, or not more than two or three young at a birth, call for remark. In such as have a numerous litter, as the sow, the young are individually so small, relative to the size of the mother, as to preclude almost the possibility of causing much difficulty in the birth. Notwithstanding this, I have known parturition fatal both to the cat and the bitch, which, as you know, have a numerous offspring.

It may, I think, be regarded as a law, that the paturient act, in domesticated animals, is easy or otherwise, in proportion as they are subjected to a more or less laborious life. Hence the mare, which is seldom permitted to be idle, rarely dies in parturition. It is in the cow and the sheep, particularly the former, that the act of bringing forth their young is so often attended with difficulty, and even with fatal consequences. In country dairies, where the cow is daily abroad in the open air up to the period of calving, and feeds upon herbage, parturition is comparatively safe and easy; less so, however, I am inclined to think than in mankind; but in town dairies (you are aware that in most great towns very large dairies are kept,) the act of parturition is incredibly dangerous, so much so that it is seldom the dairy proprietor chooses to keep the same cows for more than one year. During each season he sells off his stock, and supplies their places with cows in-calf, purchased from the country farmer; and these he does not admit into the cow-house till they are within eight or ten days of the period of calving. When, on account of her good qualities, he is induced to retain a milch cow year after year, the risk in parturition, and from its consequences, is reckoned equal to one fourth of the value of the animal.

Thus we find that in town dairies, where the state of the cow is wholly artificial, (that is, where she is never turned out to take either air or exercise, and is fed not on herbage, but chiefly on warm boiled grains,) parturition is attended

* The individual alluded to was for some time the superintendent of a dairy in the neighbourhood of Edinburgh, consisting of about three hundred cows.

with extraordinary risk; a risk fiftyfold greater than in the human kind, even under the most unfavorable circumstances that are known.

The next arguments on which the opponents of midwifery, as a science, found their objections, is the ease and safety of labour among such savages as the American Indians and the New Hollanders, where, say they, men-midwives are unknown, and woman, like every other animal, brings forth her young by the aid alone of that all-sufficient midwife, nature. Whether or not it be true that women, in this state of society, bear children with more safety than those of our country, we shall presently inquire. I readily admit, however, that the women of savages experience less pain during parturition, and, comparatively, still less danger and suffering in the puerperal state, than the women of civilization. This is owing to a variety of causes which may easily be pointed out. In a rude condition of society, a great proportion of females, and especially such as are feeble and deformed, are destroyed in infancy. This practice obtains, more or less, in perhaps every tribe of barbarians of whose manners we possess any knowledge. Hence, of course, the healthy, vigorous, and well-formed alone are reared, and live to become mothers. The life also of women, in such a condition of society, is incredibly hardy and toilsome; and this, by at once invigorating the organic frame, and limiting and repressing the influence of the mind on the body, renders them of a singularly irritable constitution, almost in the degree of wild animals. Far less sensible to pain than the civilized portion of their sex, they speedily recover from severe wounds, and other bodily injuries, with little or no sympathetic fever. It is of such women as these that James, the scientific narrator of the American expedition from Pittsburg to the rocky mountains (when speaking of the Indians in their native wilderness) remarks: "During pregnancy the squaw continues her usual avocations, and, even in its most advanced state, she neither bears a lighter burden on her back, nor walks a shorter distance in a day, than she otherwise would. If on a march she feels the pains of parturition, she retires to the bushes, throws her burden from her back, and, without any aid, brings the infant into the world. After washing in water, if at hand, or in melted snow, both herself and the infant, she immediately replaces the burden upon her back, weighing perhaps between sixty and one hundred pounds, secures her child upon the top of it, protected from the cold by an envelope of bison robe, and thus hurries on to overtake her companions."

If we are to believe many who have lived among barbarians, and written of their habits and peculiarities, childbirth in them is uniformly easy and expeditious; nearly as much so as the performance of the simplest of the animal functions. A considerable variety of recent, credible, and highly valuable evidence on this point, however, (furnished chiefly in casual hints and allusions, which is by far the most unexceptionable kind of evidence,) leads me to form a very different conclusion. So far is parturition from being easy, expeditious, and safe, in every instance, we have reason for thinking that truly difficult labours are proportionably as numerous with them as in our own country. Where there is no malposition of the fœtus or other impediment, no doubt parturition is a lighter affair with the barbarian than with the European. Of this, speaking generally, there can be no doubt; and the reason I have already briefly stated: but, in exemption from the usual causes of impeded labour, properly requiring the aid of science for the safe and speedy delivery of the patient, I believe there is either no difference at all, or, if there be, it will be found in favor of the greater exemption, from such causes, of women in a state of civilization. The blows and other ill usage in various ways which the women experience at the hands of their husbands, and the heavy burdens which they are in the daily habit of carrying up to the last hour of utero-gestation, cannot, in all cases, fail in producing either malposition of the fœtus, or injury to the connexion between the fœtus and the mother. Besides, although it be true that few instances of decrepitude, or of other defect in the bony system, are found in a rude state of society, yet there is no doubt that such defects do exist. M. Rollin, surgeon, in the Voyage of La Perouse, in his account of the physical peculiarities of the natives of California, assures us that he did not see among them one instance of rickets; and a similar remark is made by many observers regarding other barbarians: but what of this? A traveller might visit, and attentively survey, an extensive district of our own country, and behold a far more numerous population than M. Rollin, and the others I have alluded to, saw, and yet, probably, not discover a single case of hunchback, or rickety deformity. As a set-off to such sweeping observations of travellers, I may mention a few facts that are brought out, incidentally, in the narratives of some of the most credible authorities we possess, respecting the ruder portion of our species. Thus, when describing the wild tribes bordering on the Rocky Mountains, Mr. James informs us that in the Oto nation they saw a deaf and dumb boy; an adult with a curved spine; and another with an inflexible knee, the leg forming a right angle with the thigh. Among the the Kaskias they saw likewise one old woman with a distorted spine, who, Mr. James remarks, when young, had probably suffered from rickets. Scrofula, the party found, was not uncommon. In Captain Lyon's Journal of his Voyage to the North Pole, mention is incidentally made of an Esquimaux boy, of five or six years old, who, in addition to mental imbecility, was dumb, had rickets, and epileptic fits. In the account of the missionary voyage of the ship Duff to Otaheite, (this was at a period when the islanders had as yet borrowed nothing from civilization,) the writer remarks, that a person knock-kneed or bow-legged was rarely to be found. His party saw only three hump-backed children in the whole island, and they were boys. It would, of course, be vain to conjecture how many deformed girls the barbarity of the natives may have consigned to the grave, beyond the observation of the missionaries. In speaking of the natives of Tongataboo, Captain Cook remarks, that they have few natural deformities. He, however, saw two or three with the feet bent inward. By a little more industry in searching for similar facts, others, I dare say, might be discovered; but these are sufficient to demonstrate the probability, that distortion of the pelvic bones may exist in women of even the rudest tribes of whom we possess any knowledge.

But is it really true that barbarian women have always easy and safety parturition? The contrary, I am inclined to think, will be found true. I have already admitted they suffer very little from the effects of labour, just as they would suffer little from a severe accident, or a surgical operation. But in a hundred instances of labour among the Indians of the Rocky Mountains, or the New Hollanders, I see no reason to conclude that the proportion of preternatural cases would be found to be smaller than in Europe: perhaps it would be found to be greater. Although minute and specific information on this point is not to be attained, I have collected a number of remarks more or less directly bearing upon it. Long (a noted fur-trader), in his book entitled "Voyages and Travels among the American Indians," when descanting on the fortitude of the savages, mentions incidentally the fact of a young woman, of the Rat nation, being in labour a day and a night without uttering a groan; the force of example acting so powerfully on her pride as not to allow her to express the pain she felt. Another similar fact is stated in the Voyage of Clarke and Lewis up the Missouri. The wife of an Indian of the party was in labour, and suffering considerably, when one of the Indians gave her as a remedy some of the rattle of the rattlesnake, in powder, pretty much as we should give the ergot of rye. In Hearne's Journal of his Expedition to the Northern Ocean, he casually says, "here (mentioning the name of a place) we were detained two days, owing to one of our women being taken in labour. The instant that the woman was delivered, which was not till she had suffered for near fifty-two hours, the signal was made for moving." Mackenzie, also, in his well-known Travels in the extreme North of America, incidentally remarks, that, on a particular time, the Indian hunter attached to the party returned, after a temporary absence, accompanied by his wife, leaving behind him his mother-in-law in a helpless state, with three children, and in labour of a fourth. It came out that she had been left "in a state of great danger." In the scientific expedition to the sources of St. Peter's River, its historian, Capt. Keating, states respecting the Potawatomis, a tribe with which he associated for some time, and concerning whose manners his party gained much curious information, that labour was seldom fatal among them, but that many instances had occurred in which the child was so long in being born, that it was putrid when expelled. The same writer informs us, that, in answer to inquiries made among a tribe of Indians, called Sanks, concerning the usual duration of labour, he was told that the pains of labour continued, in some cases, as long as four days. In general, however, labour continued a very short time. From the Dacotas, another Indian tribe, the same party learned that parturition among them, though generally easy, in some cases lasted from two to four days. In Franklin's Overland Journal, we have another incidental notice of labour in an Indian. A Chipawyan woman fell in labour, in the woods, of her first child, and, on the third day after, died. In Krantz's account of the manners of the Greenlanders, we have an allusion to parturition which deserves mention. He is speaking of the opinions which these savages hold regarding the qualifications that entitle them to gain admission to heaven: among others, those, it appears, find entrance who had been drowned in the sea, or have "died in childbirth."

The next fact I shall mention has reference to a people the lowest of any in the scale of civilization; I allude to the New Hollanders. Among them a man is not permitted to approach where the act of parturition is being performed. Collins, an excellent observer and writer, informs us, that he had the curiosity to get a European woman to be present at a native labour. By this person it was remarked that, while one female poured cold water, from time to time, on the abdomen, another was busy performing some trifling charm. How long the labour lasted is not said; but after it was over, the poor woman appeared much exhausted, and fell across a fire that was in the place, happily without receiving injury.

In concluding the list of facts in support of this branch of my argument, I have the gratification to present you with what I cannot but regard as valuable and interesting information concerning parturition, as it occurs in the islands of the South Sea, privately furnished me by Mr. Ellis and Mr. Bourne, missionaries, who resided (particularly the latter) a great many years in those islands of the Pacific where missions are established. In order to elicit the information I wanted, in a clear and precise form, I took the liberty of putting in writing the following questions, which I shall give you seriatim, with the replies:

1st. Is parturition, in general, speedier, easier, and safer, in the South Seas, than it is among our own peasantry? Mr. Bourne's reply: "Parturition in the South Seas is, in general, easy, speedy, and safe, even to astonishment. Immediately after delivery, the women are generally able to arise, take their infants in their arms, and bathe themselves and infants in some neighbouring rivulet. A servant to one of the missionaries, whose business it was to catch and milk her master's goats, was confined with a child on the Sunday evening, and on the Monday morning caught and milked the goats as usual." Additional reply by Mr. Ellis: "Parturition is remarkably easy: many instances of this came under our notice. A servant of ours was engaged at washing one morning; labour came on about nine o'clock; she went home; and in the evening, walking, came again, with the child in her arms. Formerly they used, immediately after delivery, to sit upon a pile of hot stones covered with herbs, and when this had produced profuse perspiration, would rush into the sea within half an hour after. I am disposed to think civilization does make childbearing more difficult, or makes the mothers feel its inconveniences more."

2d. Have very protracted, dangerous, or fatal instances of childbearing come to your knowledge? By Mr. Bourne: "Protracted, fatal, and dangerous instances of childbearing have come to my knowledge; although such are not very frequent." By Mr. Ellis: "Protracted and dangerous labours have generally been occasioned by mal-presentations; the most have been shoulder presentations, or the protrusion of the arm."

3d. Have you observed parturition to be more or less difficult according to the state of civilization: easier, for example, in New Zealand than in Otaheite? By Mr. Bourne: "Parturition is as easy in Tahiti as in New Zealand."

4th. Do the South Sea women employ a class of females in any respect answering to that of our midwives? By Mr. Bourne: "No class of women are employed in the South Seas answering to midwives." By Mr. Ellis: "There were a number of women celebrated for their skill in aiding difficult labours, but in ordinary cases their aid was not sought."

5th. In protracted and difficult labours, what means do they use to hasten delivery? By Mr. Bourne: "In protracted and difficult labours, no means are used to hasten delivery. Every thing is left to nature. The missionaries have saved many in difficult labours, that otherwise would have died. I knew of one fatal instance, and many others would have occurred but for the missionaries." The reply of Mr. Ellis is in a trifling degree different: "In difficult labours, the patient was fixed on two stools, while a friend supported the back: or, the patient sat on the extended thighs of an assistant, while another person endeavoured by pressure, and other mechanical means, to promote delivery. We have reason to believe several must have died in childbirth but for the aid of the missionaries."

After so copious an induction of facts, I need say little more, I presume, to convince you that the act of parturition, among the women of rude nations, is not, in the absence of obstetric science, uniformly safe and easy: but, on the contrary, notwithstanding the degree of perfection in which the organic functions in general, and of course the uterine, are performed in them (in which respect they are more favored than the women of civilization), protracted, and even fatal labours are at least equally numerous as they are with us.—*Med. Gazette.*

---

### MIDWIFERY.

### *Rupture of the Membranes, for the purpose of bringing on Labour before the full Period of Utero-Gestation, in Cases of Deformity of the Mother. (Gazette Médicale.)*

The question has often been discussed, whether, when women are so deformed that it would be impossible to deliver them at the full period of pregnancy, it would not be more prudent to bring on labour at the eighth month. In 1756, the most celebrated accoucheurs of London consulted upon this subject, and determined the question in the affirmative. Since that period the membranes have been frequently ruptured with a trocar, in order to bring on labour, in England, Holland, Italy, Germany, and France. After the rupture of the membranes, labour comes on, and the management of it is conducted in the ordinary way. In seventy-four cases, Reisinger met with only one which proved fatal to the mother; forty-four of the children were born alive. In Germany and Holland, out of thirty-four cases, only two women died; and the fatal result in these instances did not appear to depend upon the effect of rupturing the membranes: nineteen of the children survived. In Paris, in 1829, six labours were thus brought on; all the women did well, and only one child was lost. There can be no doubt that the operation of rupturing the membranes offers the mother a much greater chance of safety than any other means we could adopt, when the pelvic aperture is so contracted as to prevent delivery at the full term of pregnancy.—*Dict. de Médécine, from an article by* Mr. Dezeimeris.

### TOXICOLOGY.

*Curious Case of Poisoning with Arsenic.* From Dr. Elliotson's Lectures.

It is this peculiar effect of arsenic on the eyes which made me suspect the other day that a whole family had been poisoned with arsenic. I was consulted on a very curious set of cases. A whole family was seized with nausea and vomiting, and every one of them had watery eyes. This continued some time; I found all the maid-servants with watery eyes, and that they had had nausea and vomiting. There were four or five children also in the same state, as also was the lady of the house. The husband happened to be out of town. None of them had a pulse under 120, and in some it was as high as 160. The children were all going about the house, and the servants were engaged at their work. The man-servant, as well as the maids, all had rapid pulses with watery eyes. On looking at their tongues, I found they were red and foul, and they had also had thirst, and a sensation of heat at the stomach. I endeavoured to ascertain whether persons in the neighbourhood were in a similar condition, but that was not the case, not even with their nearest neighbours, and therefore I presumed the affection was not owing to the atmosphere. Then respecting the ingesta, I could discover nothing that was at all capable of explaining the symptoms. I found that they all had the general heat of the body which I have mentioned, as well as of the stomach and eyes; in fact, a general inflammatory state.

Seeing these things, and finding that the watery state of the eyes and the nausea had been going on day after day, I suspected that they were under the influence of arsenic. When arsenic produces those constitutional effects which it is necessary to remedy, you find bleeding by far the best method that can be adopted. When arsenic has been taken into the stomach, you endeavour to get it out as well as you can, but after that it is necessary to bleed on account of the gastritis, and the inflammatory state of the system. When you have got it all out of the stomach of the patient, there may still be the greatest danger, not only from the inflammatory state of the stomach, but the general inflammatory state of the body: the great remedy for this is bleeding. I felt convinced that this family must have suffered from arsenic, and they were bled, and some proportionately relieved.

The water which they drank was examined by several eminent chemists, and no arsenic could be found in it, but the persons who had lived in the house before had been mixers of colours, and some of their combinations contained arsenic; and, before they quitted the house, it turned out that they had deposited in the kitchen, and in the garden all round the house, a great quantity of arseniate of copper. I did not learn this at first, and I could not imagine where the arsenic came from; but when the husband returned home and stated this, on relating the case to an eminent chemist, he told us that he was satisfied that water coming to the arseniate of copper would produce arseniuretted hydrogen, which was let loose, and produced the effect on the family. He considered that the arseniate of copper was calculated to become pernicious by water being mixed with it. The situation of the house was damp, and water had free access to the arsenic contained in the colouring matter, which had been deposited only last summer under the kitchen and every part of the house. Several barrows full of this stuff were removed. It was an eminent chemist who informed us that, under all the circumstances, he had no doubt that arseniuretted hydrogen had been disengaged, and I had felt satisfied, from the effects I saw, that arsenic must have been the cause of all the symptoms. No medicine was given, nor was anything of the kind required, as the arsenic was not in the stomach, but had pervaded the whole system. It was altogether an extraordinary occurrence, and I never witnessed anything of the kind before. The whole family were affected. All had a pulse from 120 to 160, and the whole of them were affected with a running of the eyes, but they all got perfectly well, except that pain in the limbs came on after they had appeared well for some time.—*Lancet.*

